# The Optical Properties of Metal-Free Polymer Films with Self-Assembled Nanoparticles

**DOI:** 10.3390/polym13234230

**Published:** 2021-12-02

**Authors:** Chung-Cheng Chang, Kwang-Ming Lee, Chia-Hong Huang

**Affiliations:** 1 Department of Electrical Engineering, National Taiwan Ocean University, Keelung 202301, Taiwan; ccchang@mail.ntou.edu.tw; 2 Department of Chemistry, National Kaohsiung Normal University, Kaohsiung 824004, Taiwan; kmlee@nknu.edu.tw; 3 Department of Electronic Engineering, National Kaohsiung Normal University, Kaohsiung 824004, Taiwan

**Keywords:** polymer films, antireflection, self-assembled nanoparticles, light scattering, wide band-gap

## Abstract

In this paper, it is reported that a metal-free and non-conjugated polymer, MA-PEG 8000-BADGE (MP8B), exhibits an antireflective property and substrate-dependent photoluminescence (SDP). MP8B was constructed from maleic anhydride, poly(ethylene glycol) and bisphenol-A diglycidyl ether. Self-assembled nanoparticles are found in MP8B and can prospectively act as scattering centers to improve light trapping and extraction. MP8B films prepared from MP8B solutions have been characterized by photoluminescence (PL), atomic force microscopy (AFM), tunnelling electron microscope (TEM), reflectance, transmittance, and UV-Vis absorption spectrum. MP8B films can suppress light reflection and enhance light transmission. The PL spectrum of MP8B film on ITO peaks at approximately 538 nm, spanning from 450 to 660 nm at a concentration of 25 mM. Meanwhile, the effects of concentration and substrate on the PL of MP8B films are also investigated in this study. Surface roughness becomes larger with concentration. A red shift of the PL spectrum is observed as solution concentration increases. Meanwhile, aggregation-caused quenching (ACQ) is insignificant. Moreover, the PL spectra of MP8B films show a substrate-dependent phenomenon due to dielectric screening. The optical band-gap energy of MP8B is approximately 4.05 eV. It is concluded that MP8B is a promising candidate for a host material, and its film can be utilized as a multifunctional layer (i.e., antireflective and light-scattering functions) for optoelectronic applications.

## 1. Introduction

Optoelectronic devices, such as solar cells, light-emitting diodes (LEDs), photodetectors, and laser diodes, possess multilayer structures. Therefore, significant light reflection occurs at the interfaces because of a refractive index mismatch between two media. Unfortunately, reflection causes light loss so that optoelectronic devices have an impact in performance. For instance, most of the generated photons are trapped within organic light-emitting diodes (OLEDs) at the substrate-air interface, in the transparent anode/organic layer, and at the cathode-organic interface owing to total internal reflection and evanescent coupling [1]. As a result, OLED usually exhibits an external quantum efficiency (EQE) of only approximately 20% without utilizing light extraction (high transmission) techniques [2]. Similarly, a polymer bulk heterojunction solar cell reflects approximately 30% of the light reaching the surface of the solar cell without using antireflective (AR) technologies [3]. Hence, AR technologies are an important issue that can be utilized to eliminate reflection and improve device performance in photovoltaic (PV) [4,5,6,7,8,9,10], lighting [9,11,12,13,14,15], displays [16,17,18], photodetectors [18,19], and laser [20,21,22,23] applications. Antireflective coatings (ARCs) are usually made of single-layer [24,25,26,27], multilayer [28,29], or micro/nanostructured [30,31,32,33,34,35,36,37,38,39,40,41,42,43] coatings. Single-layer ARCs are facile but cannot suppress reflection in a broadband solar spectrum [4], whereas multilayer ARCs are complicated to produce because of the requirements of the refractive index and the control of layer thickness. Furthermore, thermal mismatch induced strain and material migration of multilayer ARCs degrade the device performance in high power operation [42]. 

An alternative to multilayer ARCs are micro/nanostructured ARCs with structure dimensions comparable with light wavelength. Micro/nanostructured ARCs are manufactured by different technologies that involve photolithography [9,18], imprinting [7,17,39], molding [10,30], etching [40,41,42], or vacuum processing [14,34]. However, these technologies are considerably complex or expensive for large scale and mass production. Therefore, there has been much interest in polymeric thin films produced by wet-processable and scalable techniques thanks to their simple and cost-effective methods. Similarly, micro/nanostructured antireflective polymeric thin films are also prepared by nanopatterning methodologies, such as etching [7,10,40], imprint lithography [17], and microinjection compression molding [30], which are not simple or cheap, as mentioned above. In our previous report, we synthesized the non-conjugated polymer without using organometallic reagents and solvents [44]. Self-assembled nanoparticles were observed in the polymer. It was proposed that self-assembled nanoparticles are produced in a synthesis process with bisphenol-A aggregates and poly(ethylene glycol) moieties. In addition, the average size of the self-assembled nanoparticle in the polymer film increases with the concentration of the polymer solution [45]. Self-assembly is a cost-efficient and high-throughput approach for manufacturing nanoscale architectures. In this study, MP8B polymer synthesized by an environment-friendly method without utilizing any solvent is low-toxic and bio-compatible, and its film, which is prepared by spin coating, exhibits antireflective and high-transmission properties and substrate-dependent photoluminescence (SDP). Self-assembled nanoparticles are also observed in MP8B films so that the production of MP8B film with self-assembled nanostructures is low-cost. In particular, the major advantage of such disordered nanostructures is that the spectrum is independent of viewing or incidence angle. Furthermore, it has been shown in the literature that thin films with nanoparticles can enhance light extraction and transmission for optoelectronic applications [27,46,47]. Moreover, scattering layers with nanoparticles are industrially relevant candidates that can improve the light outcoupling of OLED [1]. Thus, MP8B films can prospectively act as scattering layers to improve light trapping and extraction for optoelectronic applications. On the other hand, conjugated polymers have not only raised concerns regarding environmental issues but they also often suffer from aggregation-caused quenching (ACQ), [48] so they are significantly restricted for optoelectronic applications. However, the emission of MP8B film is not significantly quenched in this study. Meanwhile, a red shift in PL and UV-Vis absorption spectra is observed with solution concentration. Furthermore, the optical band-gap energy (*E*_g_) of MP8B film is approximately 4.05 eV. It is, therefore, expected that MP8B polymer can not only be applied to ARC and light transmission but can also be used as a host material because of its metal-free and wide band-gap properties for multifunctional optoelectronic applications.

## 2. Materials and Methods

### 2.1. Synthesis of Polymer

The polymer was synthesized according to the method reported in the literature [44]. All chemicals were purchased from commercial suppliers and used as received without further purifications. First, 1 mmol of Maleic anhydride (CAS No. 108-31-6) and 0.5 mmol of poly(ethylene glycol) 8000 (CAS No. 25322-68-3) were mixed and stirred under N_2_ atmosphere at 90 °C for 1.5 h without using any organic solvent. The product was recrystallized twice from ether, followed by the addition of bisphenol A diglycidyl ether (1.57 g, 4.60 mmol, CAS No. 1675-54-3). The mixture was stirred at 180 °C for 3 h in N_2_ ambient, and was then returned to an ambient temperature. Finally, a brown product was obtained and named MA-PEG 8000-BADGE (MP8B). All synthesized processing was at a pressure of one atmosphere.

### 2.2. Thin-Film Preparation

MP8B polymer was dissolved in tetrahydrofuran (THF). MP8B films prepared from MP8B solution were spin-coated for 40 s with 2000 rpm on ITO-coated glass, Si, and TiO_2_-coated Si substrates, and then annealed at 80 °C in N_2_ ambient for 1 h. Moreover, to confirm the influence of ACQ on the light emission of MP8B films, the samples prepared from using various MP8B solutions (1, 5, 10, and 25 mM) were spin-coated for 40 s at 2000 rpm on ITO-coated glass substrates, and then annealed at 80 °C in N_2_ ambient for 1 h.

### 2.3. Characterization

The thicknesses of MP8B films prepared from different solutions (1, 5, 10, and 25 mM) were measured by surface profiler (Bruker Dektak XT, Billerica, MA, USA) and were 63 nm, 65 nm, 81 nm, and 84 nm, respectively. The morphology of the polymer films were observed by atomic force microscope (Bruker, Dimension ICON, Tucson, AZ, USA). For the analysis of self-assembled nanoparticles, TEM images were captured (JEOL JEM-2100 TEM, Tokyo, Japan). PL properties were measured by a FluoroMax-4PL spectrometer (Horiba Jobin Yvon, Kyoto, Japan) and recorded in the range of 400–700 nm at 325 nm excitation wavelength at room temperature. UV-Vis absorption, reflectance, and transmittance spectra were measured using an UV/VIS/NIR spectrophotometer (UV-3150, Shimadzu Corporation, Tokyo, Japan). UV-Vis absorption spectra were also employed to estimate the optical band-gap energy (*E*_g_).

## 3. Results and Discussion

**Figure 1:**
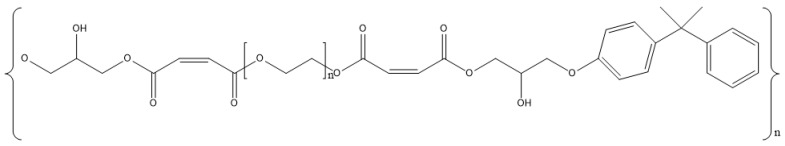
Chemical structure of MP8B polymer.

Figure 1 shows the chemical structure of the MP8B polymer. The chemical structure of the MP8B polymer was confirmed by ^1^H nuclear magnetic resonance (NMR) and IR spectra, as given in the Supplementary Materials, Figures S1 and S2, respectively. 

The elemental composition of MP8B film was investigated through XPS measurement. Figure S3 displays the XPS spectrum of MP8B film deposited on an ITO-coated glass. C 1s and O 1s peaks are obvious in the XPS spectrum. This shows that MP8B film is metal-free. Furthermore, an N 1s peak is also visible, which most likely originates from contamination. 

**Figure 2:**
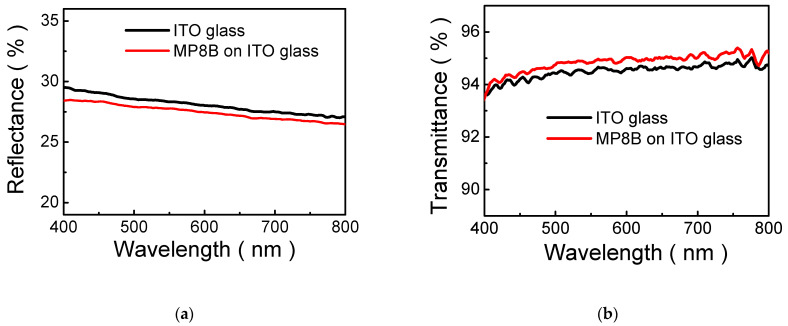
(**a**) Reflectance and (**b**) transmittance spectra of MP8B film.

To observe the optical behaviors of MP8B film, it was spin-coated onto an ITO-coated glass substrate (i.e., MP8B/ITO-coated glass). Figure 2a,b shows the reflectance and transmittance spectra of MP8B/ITO-coated glass, respectively. The reflectance of MP8B/ITO-coated glass decreases compared to that of the ITO-coated glass over a wide wavelength range of 400–800 nm, as shown in Figure 2a. This shows that MP8B film possesses antireflection properties. Meanwhile, the transmittance of MP8B/ITO-coated glass increases compared to that of the ITO-coated glass, as illustrated in Figure 2b. Notably, very little light is absorbed in the MP8B/ITO-coated glass structure (transmittance > 90%), indicating excellent optical quality. Moreover, the transmittance spectrum is relatively flat (93–95%) in the visible range, which means the optical property is insignificantly varied when it is used for lighting applications. These are attributed to the step gradient refractive-index distribution in constituent materials, i.e., air (n = 1)/ MP8B (n~1.61)/ITO (n ~ 1.82)/glass (n~1.52). In the case of a single-layer ARC, one single coating is applied on the surface of a device, such that the reflection from the air-ARC and ARC-device interfaces experience destructive interference. To achieve this and zero reflection, the optical thickness of a single-layer ARC should be equal to an odd number multiple of a quarter of the wavelength of the selected light, and its refractive index should equal the square root of the refractive index of the surface of the device. In Figure 2, the thickness of MP8B film is approximately 152 nm and is not optimized, so that the reflectance is not minimized. 

**Figure 3:**
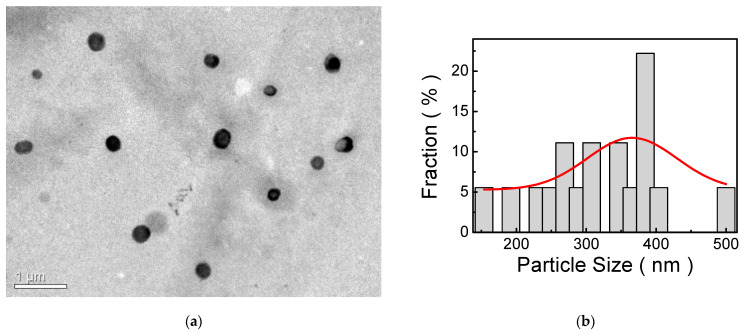
(**a**) TEM image of self-assembled nanoparticles; (**b**) The histogram of the size distribution of self-assembled nanoparticles.

Figure 3a demonstrates the TEM image of self-assembled nanoparticles. Obviously, self-assembled nanoparticles are highly dispersive, and the size of the self-assembled nanoparticle is not uniform, from 154 to 500 nm. The size distribution fitted by a Gaussian curve is also shown in Figure 3b. It shows that the mean of the size is approximately 365.7 ± 127.9 nm. Generally, self-assembly processes of organic molecules to form nanostructures are developed in solution. Therefore, it has been suggested that the nanoparticle is produced in a self-assembly process with bisphenol-A aggregates and poly(ethylene glycol) moieties serving as core and shell compartments, respectively [44]. On the other hand, the nanoparticles in the film are able to scatter the incident light [46]. Therefore, scattering caused by nanoparticles is more efficient than absorption for light trapping in photovoltaic devices [49]. In addition, the larger the nanoparticle size, the more scattering [46]. It has also been published in the literature that the size of the self-assembled nanoparticle increases with the concentration of the polymer solution [45]. Similarly, it is expected that the size of the self-assembled nanoparticle can be tuned by the concentration of MP8B solution. However, the introduction of larger nanoparticles in the antireflective (AR) film leads to a further increase in the thickness of the AR film so that the absorption of the AR film increases correspondingly. Thus, the control of the diameter of the nanoparticle is an important issue. So far, many nanoparticles have been developed, which can be categorized into metallic and dielectric nanoparticles. Although metallic nanoparticles are strong light scatterers at wavelengths near their resonant frequency, their intrinsic losses are significant. Furthermore, for metallic nanoparticles, the resonant scattering is only dominated by the electric-type resonances, whereas dielectric nanoparticles have both electric and magnetic dipole resonances simultaneously excited inside the same particle by light incidence [50]. Thus, dielectric nanoparticles are more suitable for light scattering for photovoltaic applications.

**Figure 4:**
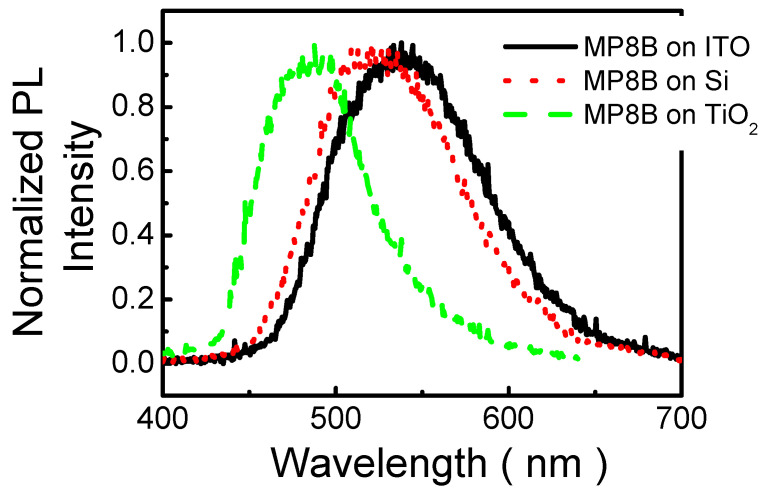
Room temperature PL spectra of MP8B films deposited on different substrates.

Figure 4 depicts PL spectra of MP8B films spin-coated on ITO glass, Si, and TiO_2_-coated Si substrates at an excitation wavelength of 325 nm. The PL peaks of MP8B films on ITO, Si, and TiO_2_ are 538, 522, and 488 nm, respectively. The PL spectra of MP8B films spin-coated on Si and TiO_2_ exhibit blue shifts (16 and 50 nm, respectively) compared with that on ITO. It has been shown that the PL of nanostructures is affected by doping [51,52,53,54,55], strain [54,56,57,58,59,60], and dielectric screening [61]. MP8B films are annealed at 80 °C after spin-coating so that the doping effect on the PL of MP8B films is not significant in this study. Moreover, the lattice constants of ITO, Si, and TiO_2_ are 10.12 Å, 5.43 Å, and 4.59 Å, respectively. Thus, the induced strain of MP8B film on ITO is less than on Si and TiO_2_, which arises from a relatively small lattice mismatch between MP8B film and ITO. Therefore, if PL is dominated by lattice mismatch induced strain, a red shift in PL spectra should be observed with increasing strain [56,57,58,59]. However, this is contradictory to our observations in Figure 4. Furthermore, it has been shown that the PL may be intensively influenced by the screening of the dielectric environment [61]. In this study, it is observed that the wavelength of the peak decreases (blue shift) with the increase in the relative dielectric constant of the underlying film, from 3.4 (ITO) to 63.7 (TiO_2_). This result may be ascribed to the dielectric screening of the Coulomb interactions, which can affect the binding energies of excitons [61]. Therefore, the larger the relative dielectric constant of the underlying layer or substrate, the smaller the Coulombic interaction between electrons and holes, which thus decreases the exciton binding energy [62,63], so that the wavelength of the luminescence decreases (blue shift). Hence, if the effect of dielectric screening dominates PL, a blue shift is expected with the relative dielectric constant of the surrounding dielectrics because the PL peak energy can be estimated by subtracting the exciton binding energy from the band-gap energy, which is in good agreement with our observations. Consequently, it is suggested that the blue shift in PL results from dielectric screening in this study. 

**Figure 5:**
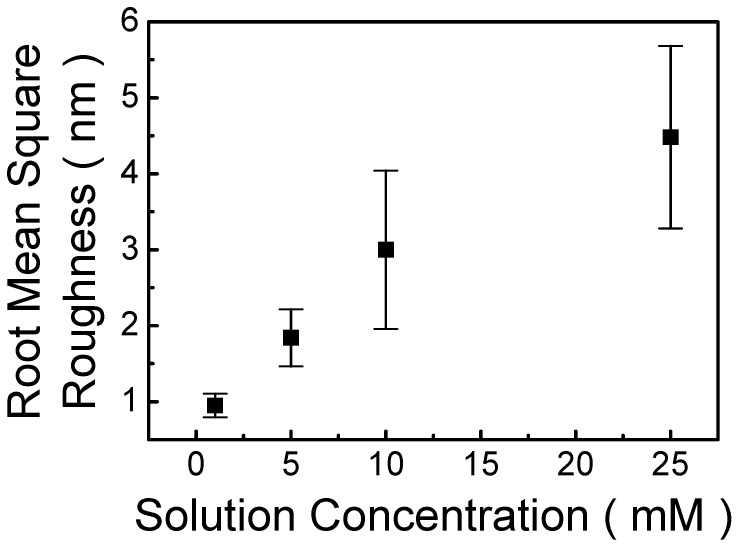
Root mean square values of MP8B films prepared from various concentrations of MP8B solutions.

The AFM images in Figure S4 show that the roughness of MP8B film changes significantly as the concentration of MP8B solution is varied. The root mean square (RMS) roughness was estimated on an area of 30 × 30 μm^2^. Figure 5 illustrates RMS roughness values vary at different concentrations. From the AFM measurements, we find the RMS roughness values, which are 0.95, 1.84, 3.00, and 4.48 nm for MP8B films prepared from 1, 5, 10, and 25 mM of MP8B solutions, respectively, as shown in Figure 5. It is proposed that the diameter of the self-assembled nanoparticle increases with the increase in the concentration of MP8B solution. This is due to the formation of the self-assembled nanoparticle from bisphenol-A aggregates and poly(ethylene glycol) moieties [45]. Therefore, the higher the solution concentration, the larger the size of the self-assembled nanoparticle, so that MP8B film is rougher at higher concentrations.

**Figure 6:**
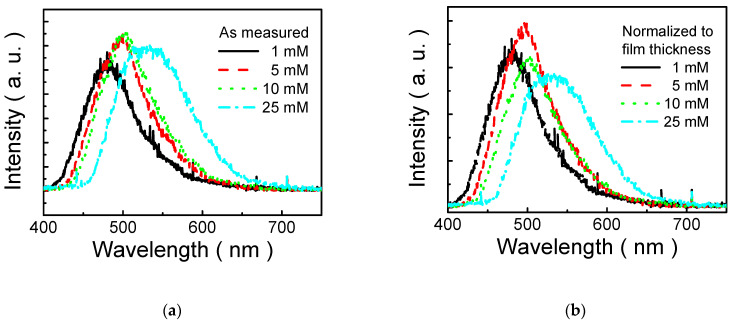
(**a**) PL spectra (original) of MP8B films prepared from various solutions (1, 5, 10, and 25 mM) and annealed at 80 °C; (**b**) PL spectra (normalized to thickness) of MP8B films prepared from various solutions (1, 5, 10, and 25 mM) and annealed at 80 °C.

Figure 6a illustrates the PL spectra of MP8B films prepared using various solutions (1 mM, 5 mM, 10 mM, and 25 mM) and annealed at 80 °C. The PL peak of MP8B film on ITO-coated glass is approximately 480 nm at a concentration of 1 mM. The PL peak is gradually changed from 480 to 538 nm when solution concentration rises from 1 to 25 mM. In other words, the PL peak shifts toward the longer wavelength (a red shift) with solution concentration. It was generally suggested that the PL peak exhibits a blue shift when lowering the size of the nanostructure due to the quantum size effect [64]. Hence, it is proposed that the diameter of the self-assembled nanoparticle decreases with a decrease in solution concentration in this study. That is, the higher the concentration of the MP8B solution, the larger the size of the self-assembled nanoparticle. This can be attributed to the larger amount of MP8B (higher concentration), which leads to more formation of bisphenol-A aggregates and poly(ethylene glycol) moieties so that the size of the self-assembled nanoparticle increases. This is consistent with the result of Figure 5.

On the other hand, it also indicates that the PL peak intensity of MP8B film is concentration-dependent. As in the cases of lower concentrations (≤10 mM), PL peak intensity increases with solution concentration. However, it slightly decreases at a concentration of 25 mM, as shown in Figure 6a. Although traditional fluorescent conjugated materials generally exhibit intensive emission in diluted solution, severe luminescence quenching of the conjugated materials is usually observed in highly concentrated solutions or in the solid state. This phenomenon is referred to as aggregation-caused quenching (ACQ) [48]. 

In general, it is known that the PL intensity depends on the sample thickness. To study the concentration dependence of MP8B luminescence efficiency in more detail, the PL intensity is normalized to film thickness to exclude the contribution of thickness shown in Figure 6b. In this way, the normalized PL spectra are not dependent on the thickness. No concentration quenching effects have been observed up to 5 mM. Unfortunately, there is a slightly downward trend in PL peak intensity at higher concentrations (≥10 mM), as illustrated in Figure 6b.

**Figure 7:**
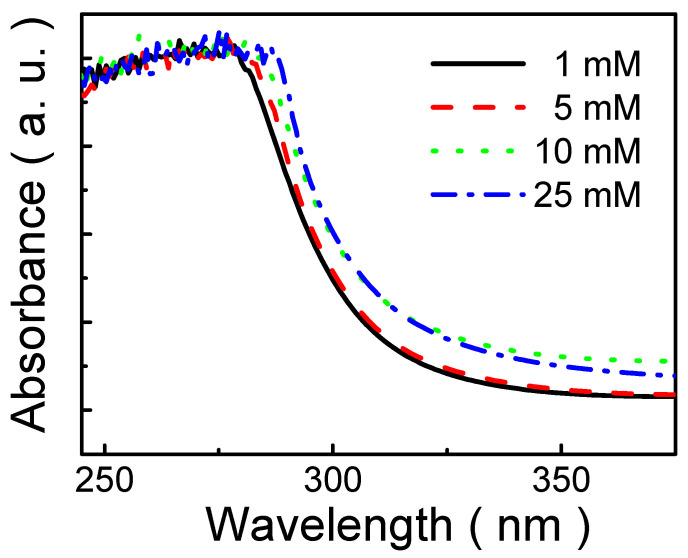
Absorbance spectra of organic thin films prepared by various solutions and annealed at 80 °C.

The UV-Vis absorption spectra of MP8B films prepared from different solutions are shown in Figure 7. It is observed that the spectra of MP8B films are red-shifted with solution concentration. This implies that more molecular aggregation and stronger intermolecular interactions are present in the polymer film prepared from the higher concentrated solutions [65]. That is, the higher the concentration, the more molecular aggregation. This is in agreement with the results shown in Figure 5 and Figure 6. On the other hand, the estimate of the optical band-gap energy (*E*_g_) is related to optical absorption edge. An exciton is generated as a photon is absorbed. The optical *E*_g_ is, therefore, derived from the onset of the optical absorption. The absorption onset is determined by linear extrapolation of the low energy edge of the UV-Vis absorption spectrum. Consequently, as solution concentrations are 1, 5, 10, and 25 mM, the onset wavelengths (λ_onset_) are 305.2 nm, 306.3 nm, 306.5 nm, and 306.8 nm, respectively. The optical *E*_g_ is evaluated by [66,67]:

*E*_g_ = 1240/λ_onset_ (1)

where λ_onset_ is the onset wavelength. Thus, the optical *E*_g_ of MP8B film decreases from 4.06 to 4.04 eV as solution concentration is increased from 1 to 25 mM. This result indicates that the optical *E*_g_ of MP8B film slightly decreases with the increase in solution concentration. Therefore, this also implies that MP8B with a wide *E*_g_ (around 4.05 eV) can be a promising candidate for a host material for polymer light-emitting diode (PLED) applications.

## 4. Conclusions

MP8B is a metal-free and non-conjugated polymer. MP8B film prepared from MP8B solution can lower reflectance and improve transmittance. The PL peaks of MP8B films on Si and TiO_2_ show blue shifts (16 and 50 nm, respectively) compared to that on ITO. The SDP is probably due to the dielectric screening in this study. The TEM image of MP8B film has shown the existence of a size distribution of self-assembled nanoparticles ranging between 154 and 500 nm. Furthermore, the RMS roughness of MP8B film becomes larger with solution concentration. It is suggested that the increase in the size of the nanoparticle with increasing solution concentration can be ascribed to the more self-assembled formation of bisphenol-A aggregates and poly(ethylene glycol) moieties in the higher concentrated solution so that the RMS roughness of MP8B film increases correspondingly. Accordingly, the control of the diameter and density of self-assembled nanoparticles is an important issue for future work. On the other hand, the PL peak exhibits a blue shift (~54 nm) with decreasing concentration, from 25 to 1 mM, owing to the quantum size effect. Therefore, it is deduced that the diameter of the self-assembly nanoparticle decreases with a decrease in solution concentration so that the PL peak shifts toward the shorter wavelengths (a blue shift) with decreasing solution concentration. This is consistent with the results of the RMS roughness. In addition, PL peak intensity is concentration-dependent. No concentration quenching effect has been observed up to 5 mM, excluding the contribution of film thickness. Nevertheless, as in the cases of higher concentrations (≥10 mM), there is a slightly downward trend in PL peak intensity. A red shift in UV-Vis absorption spectra is also observed with the increase in solution concentration. This indicates that more molecular aggregation and stronger intermolecular interactions exist in MP8B films prepared at higher concentrations. The optical band-gap energy is around 4.05 eV. These results illustrate that MP8B is a promising candidate for a host material, and its film can be utilized as a multifunctional layer (i.e., antireflective and light-scattering functions) for optoelectronic applications.

## Data Availability

The data presented in this study are available on request from the corresponding author.

## Supplementary Materials

The following are available online at https://www.mdpi.com/article/10.3390/polym13234230/s1, Figure S1: ^1^H NMR spectrum of MP8B in chloroform at room temperature; Figure S2: IR spectrum of MP8B polymer; Figure S3: XPS spectrum of MP8B film; Figure S4: The AFM images of MP8B films prepared at different concentrations of MP8B solutions: (a) 1 mM; (b) 5 mM; (c) 10 mM; (d) 25mM.
