# Supplementary material for: The Optical Properties of Metal-Free Polymer Films with Self-Assembled Nanoparticles
Source: Polymers (Basel). 2021 Dec 2;13(23):4230. doi: 10.3390/polym13234230 (PMC8659585; doi:10.3390/polym13234230)
Supplement: Supplementary file 1 [file polymers-13-04230-s001.zip › Fig S1.pdf]

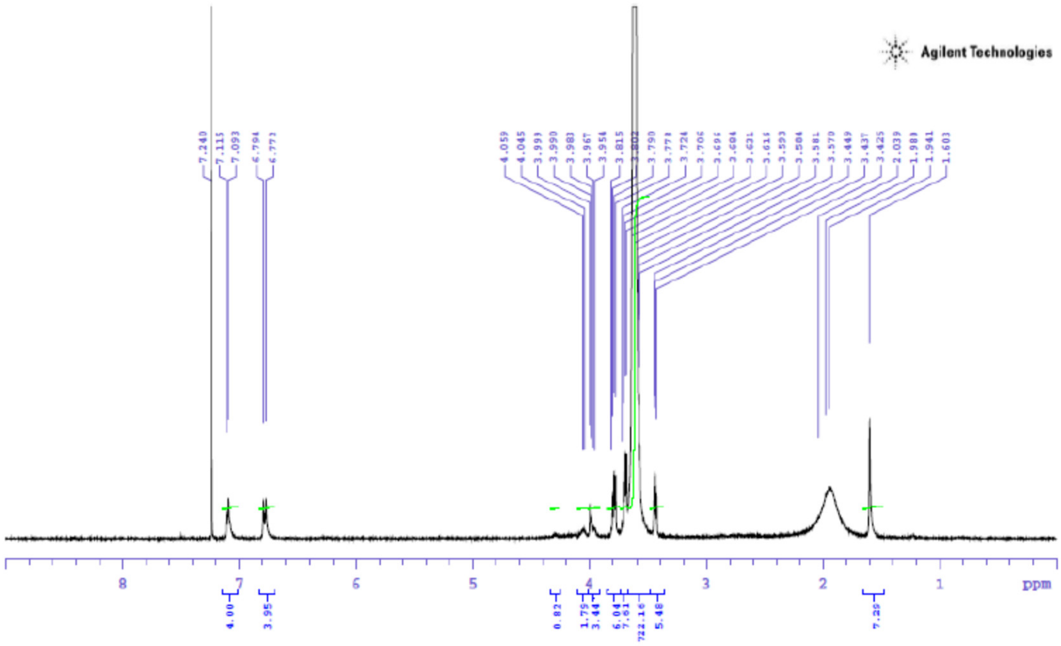

Figure S1. ^{1}H NMR spectrum of MP8B in chloroform at room temperature was recorded on an Agilent 400-MR DD2 400 MHz NMR spectrometer.
