# Supplementary material for: The Optical Properties of Metal-Free Polymer Films with Self-Assembled Nanoparticles
Source: Polymers (Basel). 2021 Dec 2;13(23):4230. doi: 10.3390/polym13234230 (PMC8659585; doi:10.3390/polym13234230)
Supplement: Supplementary file 1 [file polymers-13-04230-s001.zip › Fig S4.pdf]

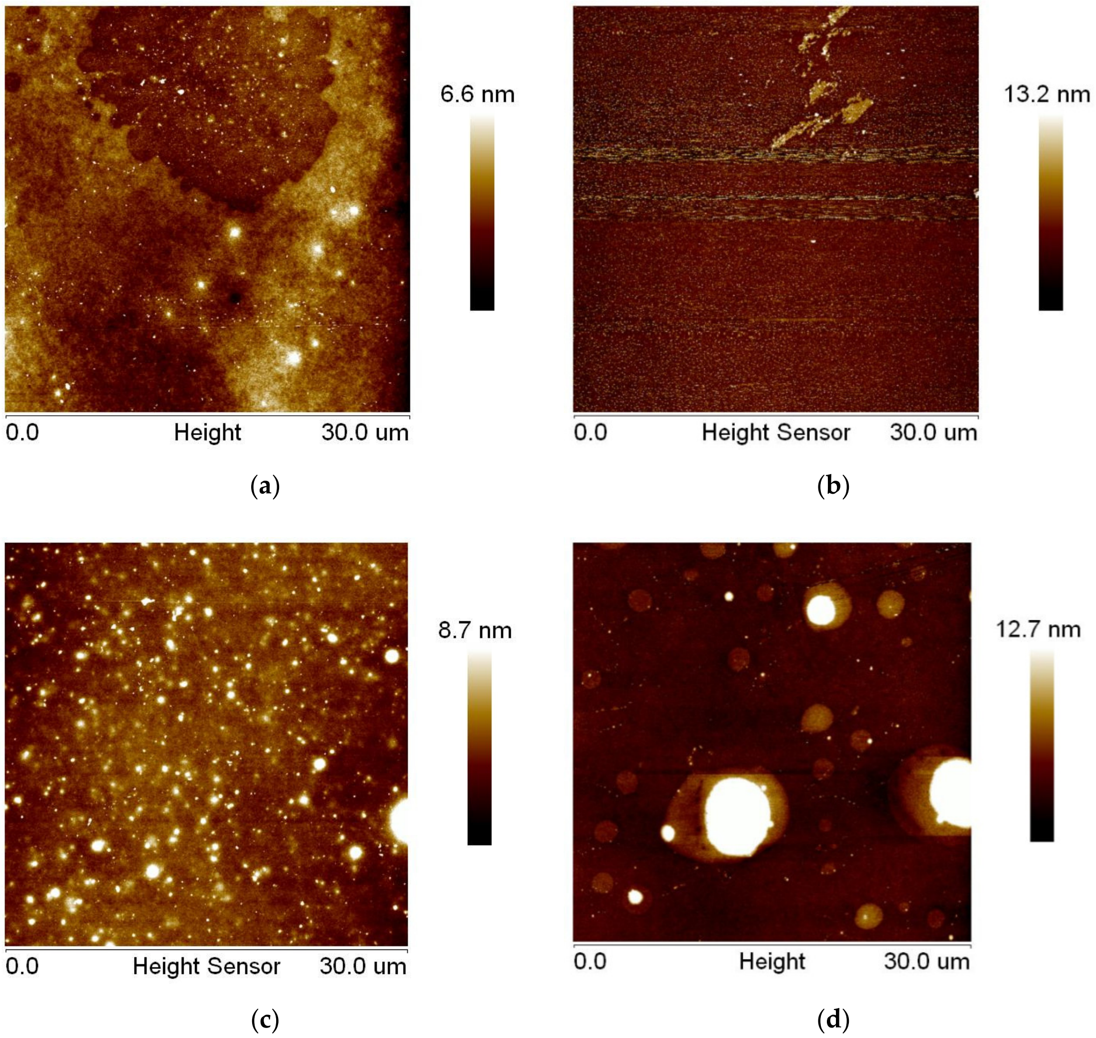

Figure S4. The AFM images of MP8B films prepared at different concentrations of MP8B solutions: (**a**) 1 mM; (**b**) 5 mM; (c) 10 mM; (d) 25mM. (Bruker, Dimension ICON, Tucson, AZ, USA)
