# Supplementary figures and images for: The Optical Properties of Metal-Free Polymer Films with Self-Assembled Nanoparticles
Source: Polymers (Basel). 2021 Dec 2;13(23):4230. doi: 10.3390/polym13234230 (PMC8659585; doi:10.3390/polym13234230)

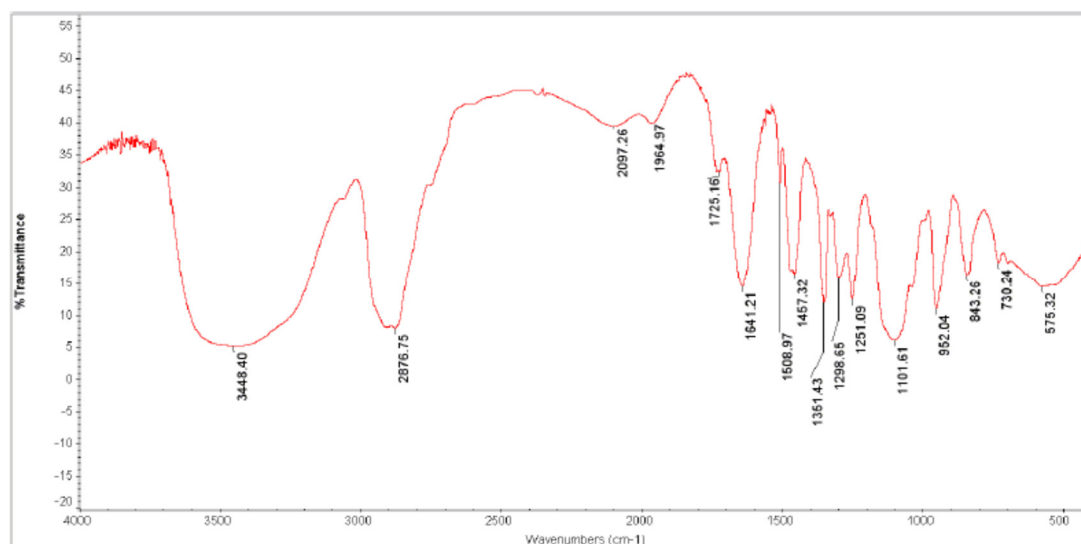

**Figure S2.** IR spectrum of MP8B polymer was recorded on a model 5700 spectrometer (Nicolet, Madison, SD, USA).

Supplement: Supplementary file 1 [file polymers-13-04230-s001.zip › Fig S2.pdf]

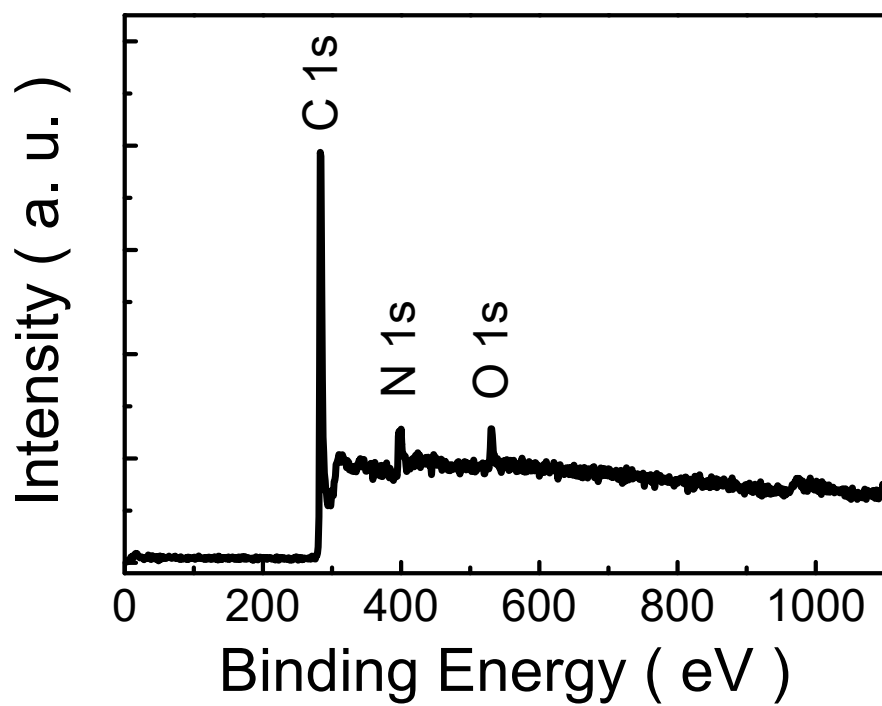

**Figure S3.** XPS spectrum of MP8B film. (ULVAC-PHI, PHI Quantera II, Kanagawa, Japan)

Supplement: Supplementary file 1 [file polymers-13-04230-s001.zip › Fig S3.pdf]
